# Multifamily QTL analysis and comprehensive design of genotypes for high-quality soft wheat

**DOI:** 10.1371/journal.pone.0230326

**Published:** 2020-03-11

**Authors:** Goro Ishikawa, Takeshi Hayashi, Kazuhiro Nakamura, Tsuyoshi Tanaka, Fuminori Kobayashi, Mika Saito, Hiroyuki Ito, Sachiko Ikenaga, Yoshinori Taniguchi, Toshiki Nakamura

**Affiliations:** 1 Division of Basic Research, Institute of Crop Science, National Agriculture and Food Research Organization, Tsukuba, Ibaraki, Japan; 2 Division of Lowland Farming Research, Kyusyu Okinawa Agricultural Research Center, National Agriculture and Food Research Organization, Chikugo, Fukuoka, Japan; 3 Division of Field Crops and Horticulture Research, Tohoku Agricultural Research Center, National Agriculture and Food Research Organization, Morioka, Iwate, Japan; Institute of Genetics and Developmental Biology Chinese Academy of Sciences, CHINA

## Abstract

Milling properties and flour color are essential selection criteria in soft wheat breeding. However, high phenotypic screening costs restrict selection to relatively few breeding lines in late generations. To achieve marker-based selection of these traits in early generations, we performed genetic dissection of quality traits using three doubled haploid populations that shared the high-quality soft wheat variety Kitahonami as the paternal parent. An amplicon sequencing approach allowed effective construction of well-saturated linkage maps of the populations. Marker-based heritability estimates revealed that target quality traits had relatively high values, indicating the possibility of selection in early generations. Taking advantage of Chinese Spring reference sequences, joint linkage maps of the three populations were generated. Based on the maps, multifamily quantitative trait locus (QTL) analysis revealed a total of 86 QTLs for ten traits investigated. In terms of target quality traits, 12 QTLs were detected for flour yield, and 12 were detected for flour redness (a* value). Among these QTLs, six for flour yield and nine for flour a* were segregating in more than two populations. Some relationships among traits were explained by QTL collocations on chromosomes, especially group 7 chromosomes. Ten different ideotypes with various combinations of favorable alleles for the flour yield and flour a* QTLs were generated. Phenotypes of derivatives from these ideotypes were predicted to design ideal genotypes for high-quality wheat. Simulations revealed the possibility of breeding varieties with better quality than Kitahonami.

## Introduction

Wheat (*Triticum aestivum* L.) is a global food crop and is consumed mainly in the form of baked products. Since high end-use quality is essential for the economic value of wheat and determines farmers' return on investment, improving end-use quality is one of the primary objectives of wheat breeding programs [1]. The wheat flour extraction rate during milling is an important end-use quality trait. However, a higher extraction rate tends to cause a darker flour color due to contamination by the bran fraction during milling. To obtain high-quality flour that is more refined and has a higher extraction rate, wheat end-users use flour color for prediction and classification purposes. It is well known that flour color is affected by protein content. Increasing the protein content of flour almost always leads to darker noodles [2]. Therefore, in breeding for improved flour quality, it is important to take milling performance, flour color and protein content into account simultaneously.

Since evaluations of quality traits are expensive and a large amount of grain is needed, such traits are not usually evaluated until late in a wheat breeding program. To overcome these problems, many studies have been performed to identify quantitative trait loci (QTLs) and associated markers for selecting favorable alleles in early generations of the breeding program (reviewed in Kiszonas and Morris [3]). Using biparental populations, the most influential QTLs were found on chromosomes 3A, 5A and 7D, accounting for 22%, 19% and 19% of milling yield variation, respectively [4]. Carter et al. [5] reported QTLs for break flour yield on chromosomes 3B and 4D. A QTL for total flour yield on chromosome 2B was previously identified as being associated with both flour yield and break flour yield [6, 7]. For flour color, polyphenol oxidase genes (*Ppo*) on group 2 chromosomes and phytoene synthase genes (*Psy*) on group 7 chromosomes have been well studied due to their relationships with the discoloration and yellowness of products, respectively [8–15]. However, QTLs containing many other genes involved in flour color-related traits have been identified [16–18]. Genetic dissection of flour protein content has been intensively performed because this trait directly affects processing quality. QTLs for protein content have been reported on 18 of 21 chromosomes [3].

High-density genotyping platforms for wheat, such as 90K iSelect [19] and 660K Axiom arrays (http://wheat.pw.usda.gov/ggpages/topics/Wheat660_SNP_array_developed_by_CAAS.pdf), enable us to perform genome-wide association studies (GWASs) using a set of germplasms or advanced breeding lines. GWASs of wheat quality traits have drastically emerged within the last five years and revealed many significant marker-trait associations (MTAs) across the wheat genome [20–25]. These MTAs would be useful for improving wheat quality. However, Kiszonas and Morris [3] pointed out in their review paper that although the wheat research community have collected numerous markers and QTLs, very few of the markers and QTLs are being used for the improvement of wheat. The low utilization of this genetic knowledge is mainly due to the lack of information on the availability of these markers and QTLs in breeding materials.

Generally, during long-term breeding programs, favorable QTLs are expected to accumulate in most breeding materials. Our previous study revealed that ongoing pyramiding of flour yield QTLs occurred during the history of wheat breeding [22]. Based on these results, pyramiding of favorable alleles from various donors into an elite variety will be continued in future breeding. Therefore, it is important to find QTLs that are not carried in an elite variety and to investigate the allelism of the detected QTLs among breeding materials. One effective way to extract useful QTL information is to perform QTL analysis jointly for multiple families derived by crossing some accessions with a single reference line [26]. In multifamily QTL analysis, information on accessions that possess QTL alleles different from those of the reference line can be obtained, which will be very useful in future breeding programs. In addition, by incorporating a variable that indicates segregation of each QTL in each family into a statistical model, it is possible to infer which of the donor lines possess QTL alleles different from those of the reference line.

Recently, the first reference genome sequences of Chinese Spring (CS) wheat were released [27]. The information provides us with a precise comparison of QTL positions among mapping populations. Furthermore, taking advantage of next-generation sequencing technology, cost-effective and robust strategies involving amplicon sequencing of multiplex samples were established for hexaploid wheat [28, 29]. These methods enable us to construct linkage maps and to perform marker-assisted selection (MAS) for a moderate number of markers in a short period of time. Since the method is flexible for selecting markers, it is possible to construct several linkage maps with as many common markers as possible very efficiently.

In this study, we used three doubled haploid populations derived from crosses between three breeding lines and a common variety, Kitahonami, which is a leading variety in the Hokkaido region of Japan. The objective of this study was to construct linkage maps of the three populations using amplicon sequencing and to determine whether common QTLs could be detected in the three mapping populations. Furthermore, we estimated the probability of segregation of each QTL in each population by a Bayesian method for jointly analyzing three populations. Based on the results, we constructed a comprehensive breeding design of genotypes that show superior quality by combining favorable QTLs from four parental varieties.

## Materials and methods

### Plant materials

Four soft winter wheat varieties, Kitahonami, Kinuhime, Shunyou and Tohoku224, were used in this study. Kitahonami was released in the Hokkaido prefecture of Japan in 2006 and has become a leading variety in Japan. The variety shows superior milling properties and high noodle-making quality. Tohoku224, which was later released as Yukiharuka, is adapted to the northeastern region of Japan, while Kinuhime and Shunyou are adapted to the central region of Japan. The last three varieties show relatively poor milling properties compared to Kitahonami. We developed three F_1_-derived doubled haploid mapping populations for genetic analysis: Kinuhime/Kitahonami (KK), Shunyou/Kitahonami (SK) and Tohoku224/Kitahonami (TK). Each population consisted of 188 lines. The four parental varieties were grown with two replicates under field conditions in Morioka, Iwate, Japan (39.7°N, 141.1°E), during four successive cropping seasons from 2010/2011 to 2013/2014. The plot size was 1.5 m x 0.7 m, and each plot consisted of 25 plants separated from each other by 12 cm. The three mapping populations were grown under the same plot size as the parental varieties but without replication. Each doubled haploid line (DHL) was subjected to a field trial for at least two cropping seasons.

### Trait evaluations

The heading dates of the accessions were recorded in all experimental plots. To compare data among cropping seasons, heading dates were converted into days to heading (DH) from May 1st of each year. Grain samples harvested from field trials were subjected to quality analysis. For each field plot, 100 grams of clean grain was tempered to a 14.5% moisture content and milled using a Quadrumat Junior instrument (C.W. Brabender Instrument Inc., South Hackensack, NJ). Milling was performed at a speed of 21.4 g/min and with a silk 72GG filter attached as a reel sieve. During the milling process, flour was divided into faster and slower halves in the flour drawer of the equipment. The faster and slower flours were called "A flour" and "B flour", respectively. Flour yield (FlYd) was calculated as the percentage of total flour weight (A flour + B flour) relative to sample weight (A flour + B flour + bran). Flour efficiency (FlEf) was obtained by the percentage of A flour weight relative to total flour weight (A flour + B flour). The A flour was subjected to an additional sieving step using a stainless steel 212 μm testing sieve (Tokyo Screen Co. Ltd., Japan). Sieved A flour was subjected to evaluation of color values using a ZE-6000 meter (Nihon Denshioku, Japan) based on 3-dimensional color values with the following rating scale: L* value (FlL) for whiteness (100: white, 0: black), a* value (Fla) for red-green chromaticity (+60: red, -60: green), and b* value (Flb) for yellowness (+60: yellow, -60: blue). A six-gram flour sample was combined with 10 ml of distilled water to form a paste, which was then mixed well without bubbling. Flour paste was poured into a Petri dish for ZE-6000 analysis, and the three color parameters were measured with the illuminant: C and angle: 2° settings. In addition, particle size distribution was measured using a HELOS Particle Size Analyzer (Sympatec GmbH, Clausthal-Zellerfeld, Germany) as follows: flour particle specific area (Sm) and median size (x50) were used as representative parameters of particle characteristics. Flour protein content (FPC) was measured using near-infrared spectroscopy with an Infratec 1241 instrument (FOSS, Hilleroed, Denmark) and adjusted to a 13.5% moisture content. Flour ash content (Fash) was determined by combustion at 600°C for 4 hours. All traits, except for the milling properties, were measured twice, and arithmetic means were used as the trait values for each sample.

### Genotyping and linkage map construction

DNA was extracted from ground leaf tissue using a PI-50α automated DNA extraction system (Kurabo, Japan). Publicly available simple sequence repeat (SSR) markers (GrainGenes 3.0, https://wheat.pw.usda.gov/GG3/) were used for screening polymorphisms among the four parental varieties using capillary electrophoresis with a QIAxcel system (QIAGEN, Hilden, Germany), and polymorphic SSR markers were used to genotype DHLs with the same equipment. To increase the number of genetic markers, approximately 3,000 originally developed amplicon sequencing markers were used (S1 Table). Based on the physical positions and genome specificity of the markers, we selected 500–600 markers for genotyping in each population. Genotyping via amplicon sequencing was performed following the protocol described in Ishikawa et al. [29]. We also used established diagnostic markers, such as *Wx-A1*, *Wx-B1*, *MFT* (*Mother of FT*), *Vp1-A1*, *Ppd-A1*, *Ppd-D1*, *Psy-B1*, *Vrn-A1* and *Vrn-D1* (reviewed in Liu et al. [30]). Linkage maps of the three populations were separately constructed with MapDisto v1.3.5 [31]. Linkage groups were identified using a minimum logarithm of odds (LOD) score of 4 and a maximum recombination fraction of 0.30. Recombination fractions were converted into centimorgan (cM) map distances using the Kosambi mapping function. Taking advantage of International Wheat Genome Sequencing Consortium (IWGSC) CS reference sequences [27], common linkage maps of the three populations were constructed. We selected 860 single nucleotide polymorphism (SNP) markers, and the physical positions of the markers were estimated by a BLAST search of marker sequences against the reference sequence. Based on the positions of the markers, the genetic distances were recalculated while considering all DHLs.

### Statistical analysis and Bayesian QTL mapping

All statistical analyses were performed using the R platform [32]. Analysis of variance and linear models were implemented with the base package of R. Significance test of correlation coefficients were performed by the ‘cor.mtest’ function in the ‘corrplot’ package. P-values of correlation coefficients were obtained via multiple comparison tests for all pairs of traits. Marker-based heritability estimation was performed using the ‘heritability’ package. To construct a relatedness matrix among DHLs, we used the ‘kin’ function in the ‘synbreed’ package with the ‘realized’ method. For each trait, the phenotypic value of each DHL was corrected by removing the effects of cropping season, and the phenotypic values were then averaged over the replicates of each DHL. This modified phenotypic value was used for QTL analysis. Three DHL populations were separately and jointly analyzed with a Bayesian QTL mapping method using the model described by Hayashi and Iwata [26]. In short, the model assumed biallelic QTLs with an allele, Q, derived from Kitahonami and an alternative allele, q, and was written as follows:

$$y_{ij}=\mu_{i}+\sum_{l=1}^{N}s_{il}u_{ijl}a_{l}+e_{ij}$$ (1)

where *y*_*ij*_ was the modified phenotypic value of the *j*th DHL in the *i*th population; *μ*_*i*_ was the mean of the *i*th population; *N* was the number of QTLs fitted in the model; *u*_*ijl*_ was a variable indicating the QTL genotype of the *j*th DHL in the *i*th population, taking a value of 1 and -1 for QTL genotype QQ and qq, respectively; *a*_*l*_ was the effect of the *l*th QTL; *s*_*il*_ was a variable indicating the segregation of the *l*th QTL in the *i*th population, with values of 1 and 0 indicating QTL segregation and no QTL segregation in the *i*th population, respectively; and *e*_*ij*_ was the residual, which followed *N*(0,*σ*_*e*_^2^), with *σ*_*e*_^2^ being the residual variance. These model parameters, including *N*, were estimated through Bayesian model fitting, where their posterior distributions were constructed with a Markov chain Monte Carlo (MCMC) procedure. Specifically, for QTL detection, a total linkage map (whole genome) was divided into equal intervals of 1 cM, and a putative QTL with some effect was located in a randomly selected interval to assess whether or not the QTL located in that interval was fitted in the model. The posterior probability of QTL existence was calculated for each interval as the relative frequency of the MCMC samples where a QTL located in the interval was fitted in the model of all MCMC samples. Accordingly, the number of QTLs, *N*, was also inferred with Bayesian estimation following Sillanpaa and Arjas [33]. For more details on the Bayesian procedure applied to model (1), see Hayashi and Iwata [26]. As a result of this Bayesian estimation, QTL information was obtained for each interval, including the posterior probability of QTL existence and the magnitude of the QTL effect, which provided evidence of the presence of a QTL in that interval. A QTL was declared significant on a chromosome when the QTL intensity, defined as the sum of posterior probabilities of QTL existence over all intervals on the chromosome [26, 33], exceeded the predetermined threshold. The threshold value for QTL intensity corresponding to a genome-wide 5% significance level was determined with 100 repetitions of permutation tests, where the maximum QTL intensity over all chromosomes was obtained by analyzing permutated phenotypic data every repetition and the fifth-highest QTL intensity value among the 100 repetitions was adopted as a threshold. When the QTL intensity of a chromosome exceeded twice the threshold, two different QTLs were assumed to exist on the chromosome, where the boundary between the two QTL regions was delimited such that the sum of the posterior probabilities over intervals in each QTL region exceeded the threshold. For each significant QTL, the estimated position and effect were calculated as the weighted averages of QTL positions (intervals) and QTL effects in a QTL region, using the posterior probability of each interval as a weight. Moreover, the posterior probability of QTL segregation in each DHL population was calculated as the relative frequency of *s*_*il*_ taking a value of 1 among all MCMC samples for each *l* (*l* = 1, 2, 3).

### Construction of the prediction model and cross-validation

To investigate the predictability of trait values from genotypes of DHLs, six-fold cross-validation was performed in the following manner: DHLs of the three populations were randomly partitioned into six equally sized groups, each including 94 DHLs. Five of the groups were used as a training set to construct the prediction model. The model was applied to predict trait values of the remaining group (validation set). This process was then repeated six times, with each of the six groups used exactly once as the validation set.

The prediction model was constructed in the same manner as adopted in QTL analysis, where the posterior probability of QTL existence and the estimate of the QTL effect were obtained in each 1 cM interval in the common linkage map with Bayesian analysis as described above. To predict a trait value of an individual in the validation set, the genotype was imputed in all intervals over whole-genome region for the individual based on the marker genotype, and subsequently, the prediction model including the posterior probability of QTL existence and estimated QTL effect in each interval was applied to the genotype. The predicted trait value was obtained by

$${\hat{y}}_{i}=\hat{\mu }+\sum_{l=1}^{m}p_{l}u_{il}{\hat{a}}_{l}$$ (2)

where ${\hat{y}}_{i}$ was the predicted trait value of the *i*th individual in the validation set; $\hat{\mu }$ was the estimate of the intercept of the model (2); *p*_*l*_ was the posterior probability of QTL existence in the *l*th interval; *u*_*il*_ was a covariate indicating the genotype of the *i*th individual in the *l*th interval, with values of 1 and -1 corresponding to the genotypes QQ and qq, respectively, with Q being an allele derived from Kitahonami and q being an alternative allele from other cultivars; and ${\hat{a}}_{l}$ was the estimated effect of QTLs located in the *l*th interval, with *m* being the number of intervals.

### Simulation of ideotypes

Here, a QTL region was redefined as a continuous chromosomal region consisting of the 1 cM intervals described above with posterior probabilities of QTL existence greater than 0.01 and the intervals with posterior probabilities less than 0.01 but surrounded by intervals with posterior probabilities greater than 0.01. Based on the FlYd and Fla QTL regions defined in such a manner, ten ideotypes with favorable genotypes for FlYd and Fla values, which were obtained for the population of DHLs derived from the same cross combinations employed here, were considered for simulation. The first ideotype (Ideotype 1) harbored favorable alleles in all target regions and was thus regarded as an optimal genotype of FlYd and Fla, while the other nine ideotypes (Ideotype 2—Ideotype 10) were genotypes with gradually loosened restraints on Fla QTLs in ascending order based on the proportions of variance explained by the QTL regions relative to total Fla variance such that the degree of accumulation of favorable alleles in Fla QTLs decreased in the order of Ideotype 1 to Ideotype 10. Different genotypes were included in an ideotype due to variable genotypes in chromosomal regions other than the fixed QTL regions. We generated 500 genotypes for each ideotype by randomly allocating the Kitahonami-type allele and alternative allele to unconstrained regions depending on the genotype in the adjacent regions, considering the recombination fraction. Generation of random genotypes was conducted by an original Fortran program. Using the results of the above Bayesian QTL analysis of DHLs, which included information on QTLs in each 1-cM-interval chromosomal region, phenotypic values of FlYd, FlL, Fla, Flb and FPC were predicted for a total of 5,000 genotypes derived from the 10 ideotypes.

## Results

### Linkage map construction

Combining SSR, amplicon sequence and diagnostic markers, the numbers of markers genotyped were 561, 686 and 691 for the KK, SK and TK populations, respectively. With the criterion of a maximum recombination fraction of 0.30, genetic maps consisting of 35, 30 and 35 linkage groups were obtained for the KK, SK and TK populations, respectively (Table 1). These maps consisted of 499 loci (555 markers) for KK, 573 (685) for SK and 598 (683) for TK with total lengths of 3,920.5, 3,493.2 and 4,718.0 cM, respectively. In all three populations, the cumulative length of chromosomes was greatest in the D genome. The number of loci per chromosome ranged from 17 (chromosomes 3A of KK, 6D of KK and 6B of SK) to 44 (5D of TK) (26.5 on average), and the average distance between loci per chromosome ranged from 3.7 cM (3B of SK) to 12.1 cM (3D of TK) (7.3 cM on average). Using primer sequences, we estimated the physical positions of amplicon sequence markers by a homology search against the CS RefSeq v1 genome sequence [27]. The physical positions of the markers revealed that the cumulative sizes of these maps reached 13.27, 13.51 and 13.24 Gb for the KK, SK and TK populations, respectively, corresponding to 94.4, 96.1 and 94.1% of the reference genome size (14.07 Gb). The D-genome maps, which tended to have low coverage due to their low polymorphism, covered 3.75 (94.8%), 3.79 (95.9%) and 3.74 Gb (94.6%) of the genomic region. The genotypes of DHLs and genetic and physical positions of each marker are shown in S1 File.

**Table 1:** Summary of linkage maps obtained using the three doubled haploid populations.

	Kinuhime/Kitahonami (KK)				Shunyou/Kitahonami (SK)				Tohoku224/Kitahonami (TK)			
Chr/Genome	No LGs	No loci	Length [cM]	cM/locus	No LGs	No loci	Length [cM]	cM/locus	No LGs	No loci	Length [cM]	cM/locus
1A	1	20	148.7	7.4	2	23	124.1	5.4	1	31	225.4	7.3
2A	2	30	189.2	6.3	2	37	156.0	4.2	3	37	200.7	5.4
3A	2	17	175.1	10.3	1	25	180.7	7.2	3	29	203.3	7.0
4A	2	25	125.3	5.0	1	19	151.8	8.0	1	26	182.7	7.0
5A	2	29	254.8	8.8	1	34	237.5	7.0	2	31	284.3	9.2
6A	2	18	107.7	6.0	2	23	94.4	4.1	2	20	127.9	6.4
7A	1	32	299.0	9.3	1	37	233.8	6.3	1	36	334.4	9.3
1B	1	22	195.1	8.9	2	18	114.8	6.4	1	25	172.4	6.9
2B	2	24	201.6	8.4	2	26	128.6	4.9	3	23	130.0	5.7
3B	4	28	113.5	4.1	2	36	133.8	3.7	2	31	207.9	6.7
4B	1	24	142.2	5.9	1	19	126.7	6.7	1	28	219.5	7.8
5B	2	26	175.8	6.8	1	29	224.7	7.7	2	29	215.5	7.4
6B	1	20	163.1	8.2	1	17	135.0	7.9	1	21	163.1	7.8
7B	1	26	177.8	6.8	2	29	142.4	4.9	1	29	274.3	9.5
1D	1	21	208.6	9.9	1	29	171.9	5.9	2	25	217.1	8.7
2D	2	22	233.0	10.6	1	32	210.0	6.6	2	29	217.0	7.5
3D	2	20	143.0	7.1	1	28	194.9	7.0	1	18	217.5	12.1
4D	1	19	162.8	8.6	1	22	121.7	5.5	1	31	250.7	8.1
5D	1	29	287.8	9.9	1	41	277.5	6.8	1	44	385.0	8.8
6D	2	17	120.0	7.1	2	28	147.9	5.3	3	25	142.3	5.7
7D	2	30	296.6	9.9	2	21	185.4	8.8	1	30	347.2	11.6
A genome	12	171	1299.7	7.6	10	198	1178.1	6.0	13	210	1558.6	7.4
B genome	12	170	1169.2	6.9	11	174	1005.9	5.8	11	186	1382.7	7.4
D genome	11	158	1451.7	9.2	9	201	1309.2	6.5	11	202	1776.7	8.8
Total	35	499	3920.5	7.9	30	573	3493.2	6.1	35	598	4718.0	7.9

### Heritability estimates of traits

For each population, the number of observations of each trait in each environment are shown in S2 Table. The total number of observations per trait varied from 1,020 to 2,018 because Fash was not observed in all four environments and some DHLs could not be measured due to an insufficient number of harvested grains. All phenotypic data are shown in S3 Table and summarized in S4 Table. Analysis of variance of each population showed that DHLs and environments had significant effects on all traits, except that environment did not affect x50 in the SK population (S5 Table). We calculated marker-based estimates of heritability for the nine traits that were observed in all four environments. The relatedness matrix of DHLs was obtained based on genotypes of 305 common markers across the three populations. The heritability of milling properties such as FlYd and FlEf was approximately 0.65, which was slightly lower than that of DH (Table 2). Among the flour color parameters, Fla showed a heritability similar to that of milling properties (FlYd and FlEf), and Flb displayed high heritability comparable to that of DH. On the other hand, FlL had the lowest heritability among the traits investigated. In this study, FPC, x50 and Sm showed moderate heritability.

**Table 2:** Marker-based estimate of heritability for each trait.

Trait	va	ve	h2	conf.int		
DH	8.953	2.666	0.771	0.730	-	0.811
FlYd	5.879	3.453	0.630	0.564	-	0.696
FlEf	20.301	10.858	0.652	0.587	-	0.716
FlL	0.065	0.276	0.191	0.119	-	0.264
Fla	0.046	0.026	0.636	0.570	-	0.702
Flb	2.055	0.658	0.757	0.709	-	0.806
FPC	0.687	0.671	0.506	0.429	-	0.583
x50	8.518	11.336	0.429	0.346	-	0.512
Sm	76883.790	74176.810	0.509	0.431	-	0.587

va: restricted maximum likelihood (REML) estimate of the genetic variance; ve: REML estimate of the residual variance; h2: plug-in estimate of heritability; conf.int: 95% confidence interval for heritability.

### Distribution of trait values

**Fig 1:**
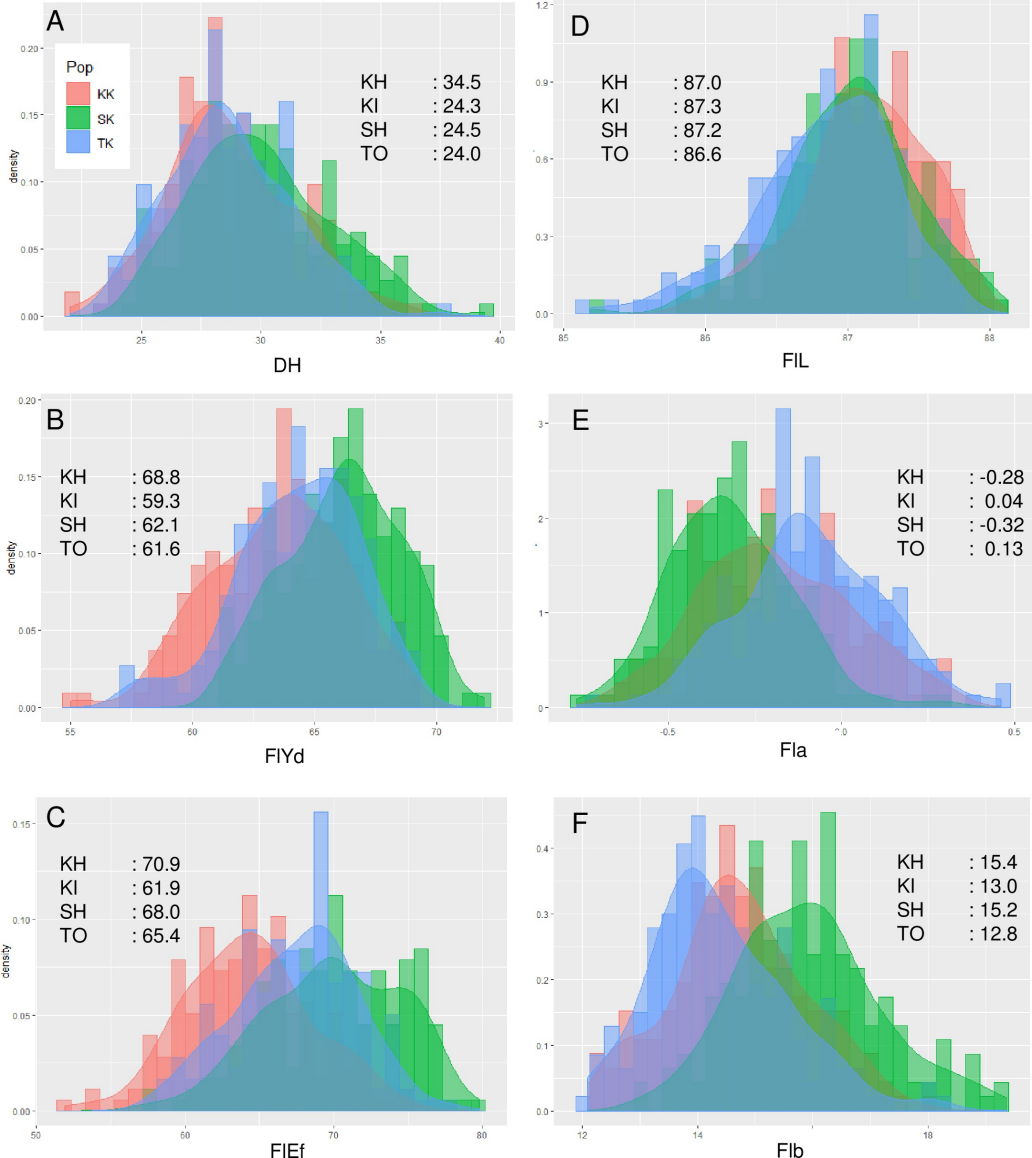
Distributions of least square means across environments. (A) DH: days to heading, (B) FlYd: flour yield, (C) FlEf: flour efficiency, (D) FlL: flour color L*, (E) Fla: flour color a* and (F) Flb: flour color b*. KH: Kitahonami, KI: Kinuhime, SH: Shunyou, TO: Tohoku224.

Phenotypic values of parents and DHLs were obtained by least square means across the four environments (S6 Table). The DH of Kitahonami (34.5) was approximately 10 days later than that of the three other parents (Fig 1A). The values for the DHLs were distributed among the parental values and showed transgressive segregation in all three populations. The phenotypic distributions of the three populations were almost overlapping. For FlYd, Kitahonami (68.8) showed the highest value, followed by Shunyou (62.1), Tohoku224 (61.6) and Kinuhime (59.3) (Fig 1B). Consistent with the parental values, the values of the SK population were higher than those of the other populations, followed by the TK and KK populations. Lines with transgressive segregation were found in the SK population, which showed higher values than the Kitahonami population. The relationship between the parental values and distribution of values in the DHL populations for FlEf was similar to that for FlYd (Fig 1C). Among the flour color parameters, the FlL values of the four parental varieties were almost the same, and the distributions of FlL values among the three populations overlapped (Fig 1D). On the basis of Fla and Flb, the four parents were divided into two groups: Kitahonami (Fla: -0.28, Flb: 15.4) and Shunyou (-0.32, 15.2) had low Fla and high Flb values, while Tohoku224 (0.13, 12.8) and Kinuhime (0.04, 13.0) had high Fla and low Flb values (Fig 1E and 1F). The distribution of values for each population included the average value of the parents as the mode, and significant transgressive segregation was observed in both directions. Distributions of the other four traits are described in S1 Fig.

### Relationships between traits

**Fig 2:**
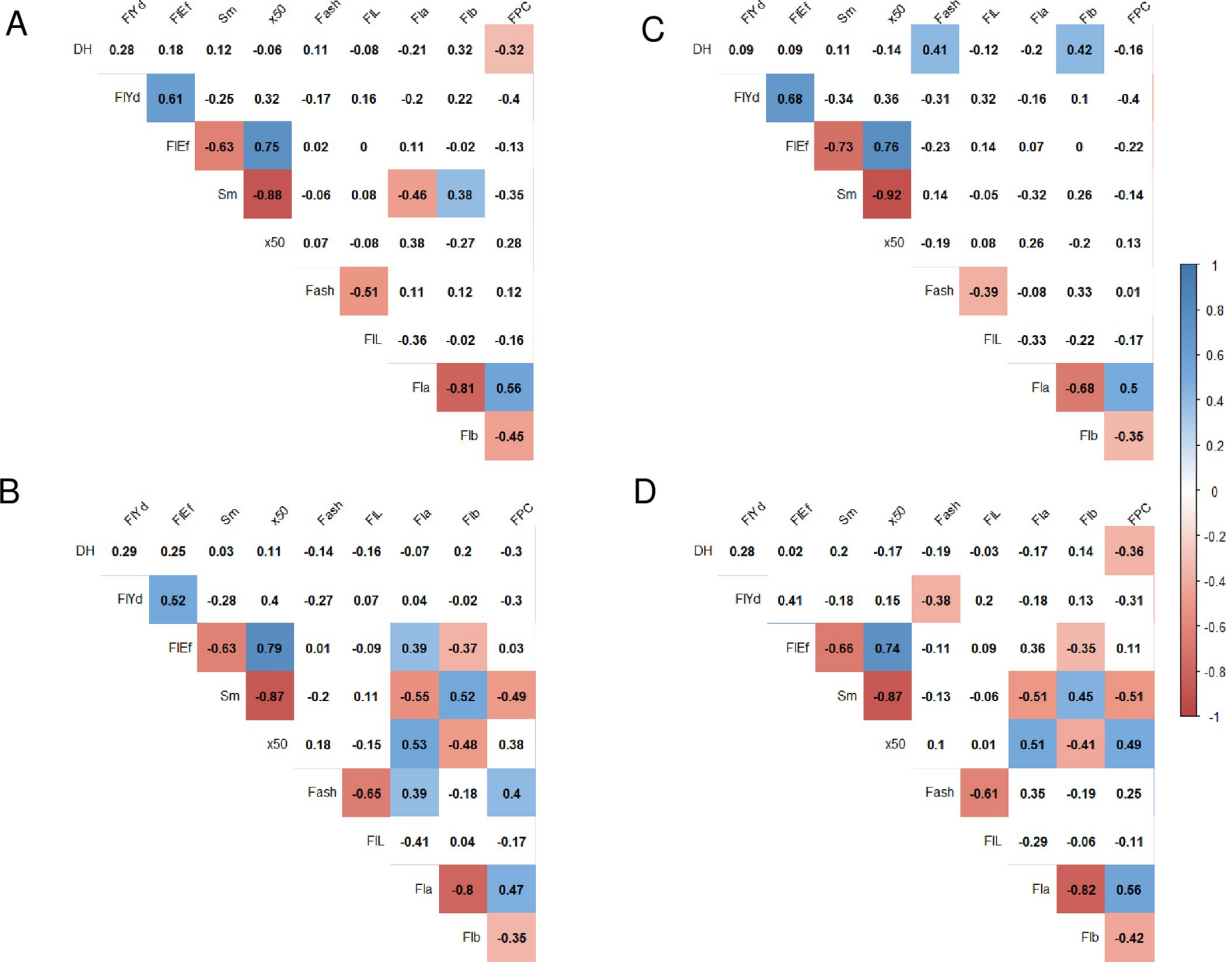
Correlation coefficients between ten traits for the three populations combined (A), Kinuhime/Kitahonami population (B), Shunyou/Kitahonami population (C) and Tohoku224/Kitahonami population (D). Colored boxes indicate that the correlation coefficients are significant at the 0.05 level. DH: days to heading from May 1st, FlYd: flour yield, FlEf: flour efficiency, Sm: specific area of flour particles, x50: median size of flour particles, Fsh: flour ash content, FlL: flour color L*, Fla: flour color a*, Flb: flour color b*, and FPC: flour protein content.

Using all DHLs, the relationships between traits were calculated (Fig 2A). In this analysis, no clear relationships were observed between DH and quality traits. A positive correlation between the milling properties FlYd and FlEf was observed (r = 0.62), and FlEf showed a strong positive correlation with x50 (0.75) and a negative correlation with Sm (-0.64). For flour color parameters, FlL was negatively correlated with Fash (-0.51). A strong negative correlation and moderate positive correlation were observed between Fla and Flb (-0.80) and between Fla and FPC (0.57), respectively. Correlations between the traits described above were also observed within the populations (Fig 2B–2D). In addition, significant correlations were observed between flour color parameters and particle size traits (x50 and Sm) in the KK and TK populations. Therefore, in the two populations, DHLs with higher Sm values had lower Fla values.

### Single-population QTL analysis

Thresholds of QTL intensity at the 5% level are shown in S7 Table. With single-population analysis of ten traits, a total of 30, 44 and 28 QTLs were detected in the KK, SK and TK populations, respectively (S8 Table). Since selection for high FlYd and low Fla values led to the development of Kitahonami, these two traits have been the focus of soft wheat breeding programs in Japan. Therefore, we mainly describe the results of these two traits in the text. For FlYd, three, six and six QTLs were found in the KK, SK and TK populations, respectively (Table 3). The QTL intensity ranged from 0.382 to 1.082 (0.763 on average). The contribution of the QTLs ranged from 0.050 to 0.133, and the cumulative contributions of the detected QTLs were 0.245, 0.589 and 0.460 in the KK, SK and TK populations, respectively. Kitahonami alleles had positive effects for thirteen of 15 QTLs. For Fla, three, four and two QTLs were detected in the KK, SK and TK populations, respectively. The intensity of the QTLs ranged from 0.499 to 1.004 (0.831 on average). The contributions of the QTLs ranged from 0.058 to 0.113, and the cumulative contributions were 0.299, 0.341 and 0.198 in the KK, SK and TK populations, respectively. In contrast to the results for FlYd QTLs, Kitahonami alleles showed favorable effects only for *QFla*.*kk*.*tarc-5D*, *QFla*.*sk*.*tarc-1B* and *QFla*.*sk*.*tarc-4A*, resulting in lower Fla values.

**Table 3:** Flour yield and flour color a* QTLs detected by single-population analysis.

Trait^1^	Pop	QTL	LG	Pos [cM]	Probability^2^	Contribution	Effect^3^
FlYd	KK	*QFlyd*.*kk*.*tarc-2D*	2D.2	87.6	0.660	0.081	0.794
		*QFlyd*.*kk*.*tarc-4D*	4D	43.2	0.392	0.064	0.740
		*QFlyd*.*kk*.*tarc-7A*	7A	136.2	0.900	0.100	0.897
	SK	*QFlyd*.*sk*.*tarc-3B*	3B.1	72.7	1.001	0.136	0.871
		*QFlyd*.*sk*.*tarc-4D*	4D	40.9	0.469	0.051	0.535
		*QFlyd*.*sk*.*tarc-5A*	5A	212.2	1.082	0.086	-0.642
		*QFlyd*.*sk*.*tarc-7A*	7A	103.9	1.000	0.133	0.860
		*QFlyd*.*sk*.*tarc-7B*	7B.1	42.1	1.001	0.118	0.826
		*QFlyd*.*sk*.*tarc-7D*	7D.1	118.7	0.577	0.064	-0.622
	TK	*QFlyd*.*tk*.*tarc-2B*	2B.2	29.3	0.382	0.050	0.568
		*QFlyd*.*tk*.*tarc-2D*	2D.2	56.2	0.388	0.059	0.621
		*QFlyd*.*tk*.*tarc-3B*	3B.1	83.6	0.997	0.092	0.778
		*QFlyd*.*tk*.*tarc-4D*	4D	114.1	0.992	0.099	0.811
		*QFlyd*.*tk*.*tarc-6B*	6B	124.4	0.682	0.076	0.691
		*QFlyd*.*tk*.*tarc-7A*	7A	198.5	0.916	0.085	0.740
Fla	KK	*QFla*.*kk*.*tarc-1A*	1A	96.1	1.001	0.113	0.083
		*QFla*.*kk*.*tarc-5D*	5D	55.2	0.765	0.084	-0.065
		*QFla*.*kk*.*tarc-7A*	7A	125.1	1.004	0.103	0.071
	SK	*QFla*.*sk*.*tarc-1B*	1B.1	34.9	0.499	0.058	-0.045
		*QFla*.*sk*.*tarc-4A*	4A	144.9	0.521	0.075	-0.050
		*QFla*.*sk*.*tarc-7A*	7A	210.6	0.866	0.096	0.058
		*QFla*.*sk*.*tarc-7B*	7B.2	26.0	0.999	0.112	0.060
	TK	*QFla*.*tk*.*tarc-1A*	1A	120.0	0.961	0.103	0.068
		*QFla*.*tk*.*tarc-7A*	7A	159.9	0.859	0.095	0.064

^1^ FlYd: Flour yield, Fla: Flour color a*

^2^ Expected posterior probability

^3^ Expected QTL effect of 'Kitahonami' allele

### Multifamily QTL analysis using CS reference sequences

**Fig 3:**
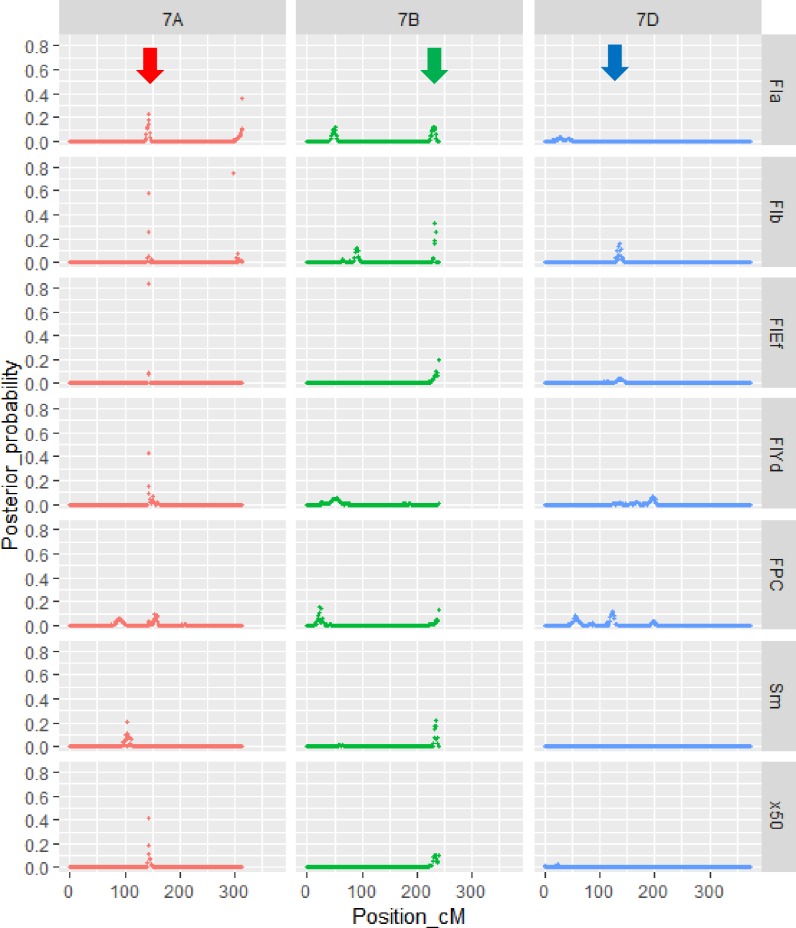
Posterior probabilities of the four traits along with group 7 chromosomes. Flour color a* (Fla), flour color b* (Flb), flour yield (FlYd) and flour protein content (FPC) QTLs were detected on group 7 chromosomes using multifamily Bayesian QTL analysis.

Based on the estimated physical positions of 860 selected markers, we constructed common genetic maps of the three populations and recalculated linkage distances (S1 File). Multifamily QTL analysis using the common maps revealed a total of 86 QTLs for ten traits. QTL intensities along chromosomes and detected QTLs are listed in S9 Table and S10 Table, respectively. Twelve FlYd and 12 Fla QTLs that exceeded the QTL intensity threshold (0.446 for FlYd, 0.344 for Fla) were detected (Table 4). For FlYd, QTL intensities ranged from 0.578 to 1.178 (0.958 on average). The contributions of the QTLs ranged from 0.021 to 0.103, and the cumulative value was 0.602. The probabilities of QTL segregation in the populations revealed that FlYd QTLs on *QFlyd*.*m*.*tarc-3B*, *QFlyd*.*m*.*tarc-4D*, *QFlyd*.*m*.*tarc-6B* and *QFlyd*.*m*.*tarc-7A* showed high segregation probabilities (>0.90) in the three populations. On the other hand, *QFlyd*.*m*.*tarc-2B*, *QFlyd*.*m*.*tarc-3A*, *QFlyd*.*m*.*tarc-5A*, *QFlyd*.*m*.*tarc-5D*, *QFlyd*.*m*.*tarc-6D* and *QFlyd*.*m*.*tarc-7B* segregated in one population. Kitahonami alleles showed positive effects for all FlYd QTLs except *QFlyd*.*m*.*tarc-3A*, *QFlyd*.*m*.*tarc-5A* and *QFlyd*.*m*.*tarc-7D*. For Fla, QTL intensity ranged from 0.436 to 1.039, with an average of 0.904. The contributions of the QTLs ranged from 0.018 to 0.081, and the cumulative contribution was 0.507. Only *QFla*.*m*.*tarc-7B*.*2* and *QFla*.*m*.*tarc-7D* segregated in all three populations, and the direction of the effect varied between the QTLs. Notably, QTL clusters were observed on group 7 chromosomes (Fig 3, S10 Table). On the 7A chromosome, *QFla*.*m*.*tarc-7A*.*1*, *Qx50*.*m*.*tarc-7A*, *QFlb*.*m*.*tarc-7A*.*1*, *QFlef*.*m*.*tarc-7A* and *QFlyd*.*m*.*tarc-7A* were detected in the 142–145 cM region. On 7B, *QSm*.*m*.*tarc-7B*, *QFlef*.*m*.*tarc-7B*, *Qx50*.*m*.*tarc-7B*, *QFla*.*m*.*tarc-7B*.*2* and *QFlb*.*m*.*tarc-7B*.*2* were located in the long arm terminal, and all of them had high segregation probabilities in the three populations. *QFlef*.*m*.*tarc-7D* and *QFlb*.*m*.*tarc-7D* were detected near *QFpc*.*m*.*tarc-7D*.*2*.

**Table 4:** Flour yield and flour color a* QTLs detected by multifamily analysis.

					Probability of QTL segregation				
Trait^1^	Chr	QTL	Pos [cM]	Probability^2^	KK	SK	TK	Contribution	Effect^3^
FlYd	2B	*QFlyd*.*m*.*tarc-2B*	119.72	0.795	0.953	0.099	0.134	0.054	0.790
	2D	*QFlyd*.*m*.*tarc-2D*	139.57	1.012	0.993	0.663	0.988	0.037	0.554
	3A	*QFlyd*.*m*.*tarc-3A*	133.00	0.682	0.727	0.674	0.987	0.021	-0.421
	3B	*QFlyd*.*m*.*tarc-3B*	98.47	1.005	0.993	0.999	0.999	0.065	0.717
	4D	*QFlyd*.*m*.*tarc-4D*	65.73	1.006	0.996	0.992	0.996	0.044	0.587
	5A	*QFlyd*.*m*.*tarc-5A*	268.17	1.138	0.447	0.984	0.140	0.050	-0.282
	5D	*QFlyd*.*m*.*tarc-5D*	261.75	1.178	0.760	0.439	0.955	0.030	0.444
	6B	*QFlyd*.*m*.*tarc-6B*	127.14	1.012	0.934	0.959	0.996	0.026	0.442
	6D	*QFlyd*.*m*.*tarc-6D*	111.45	0.578	0.995	0.036	0.144	0.103	0.952
	7A	*QFlyd*.*m*.*tarc-7A*	145.22	1.008	0.998	0.999	0.998	0.088	0.827
	7B	*QFlyd*.*m*.*tarc-7B*	59.93	1.090	0.425	0.933	0.267	0.053	0.671
	7D	*QFlyd*.*m*.*tarc-7D*	179.70	0.985	0.324	0.994	0.983	0.030	-0.497
Fla	1A	*QFla*.*m*.*tarc-1A*	125.00	1.039	0.997	0.057	0.992	0.074	0.066
	2B	*QFla*.*m*.*tarc-2B*	61.13	0.436	0.776	0.083	0.988	0.029	-0.038
	4A	*QFla*.*m*.*tarc-4A*	205.01	1.037	0.954	0.975	0.040	0.036	-0.047
	5D	*QFla*.*m*.*tarc-5D*	79.59	1.027	0.980	0.009	0.997	0.051	-0.050
	6A	*QFla*.*m*.*tarc-6A*	19.14	0.999	0.916	0.047	0.998	0.032	-0.042
	6B	*QFla*.*m*.*tarc-6B*	163.14	0.980	0.995	0.990	0.002	0.029	0.044
	6D	*QFla*.*m*.*tarc-6D*	133.75	0.682	0.988	0.208	0.669	0.028	-0.041
	7A	*QFla*.*m*.*tarc-7A*.*1*	142.21	1.003	0.999	0.254	0.998	0.081	0.063
	7A	*QFla*.*m*.*tarc-7A*.*2*	310.39	1.000	0.726	1.000	0.672	0.032	0.041
	7B	*QFla*.*m*.*tarc-7B*.*1*	50.59	1.016	0.982	0.987	0.532	0.029	-0.039
	7B	*QFla*.*m*.*tarc-7B*.*2*	229.20	1.000	1.000	1.000	0.950	0.067	0.059
	7D	*QFla*.*m*.*tarc-7D*	39.68	0.631	0.924	0.906	0.964	0.018	-0.030

^1^ FlYd: Flour yield, Fla: Flour color a*

^2^ Expected posterior probability

^3^ Expected QTL effect of 'Kitahonami' allele

### Trait predictabilities from the genotypes

The predictabilities of five traits for the DHLs were evaluated by six-fold cross-validation. The mean correlation coefficients (prediction accuracies) between the predicted and observed values of Fla, Flb, FlL, FlYd and FPC were 0.482, 0.566, 0.144, 0.485 and 0.369, respectively (Table 5). The correlation coefficients of FlYd and Fla varied among the validation sets: from 0.641 to 0.362 for FlYd and from 0.680 to 0.360 for Fla. On the other hand, the correlation coefficients of Flb ranged from 0.621 to 0.448, indicating moderately stable predictability. The predictabilities of FlL were the lowest among the five traits investigated.

**Table 5:** Correlation coefficients between predicted and observed values obtained by six-fold cross-validation.

	Validation set							
Trait	cv1	cv2	cv3	cv4	cv5	cv6	Mean	S.D.
Fla	0.574	0.397	0.360	0.680	0.489	0.393	0.482	0.114
Flb	0.579	0.599	0.448	0.621	0.596	0.555	0.566	0.057
FlL	0.064	0.094	0.123	0.249	0.141	0.191	0.144	0.061
FlYd	0.564	0.479	0.641	0.377	0.362	0.489	0.485	0.098
FPC	0.260	0.375	0.409	0.308	0.402	0.458	0.369	0.066

S.D.: Standard deviation

### Construction of ideal genotypes and prediction of trait values

**Fig 4:**
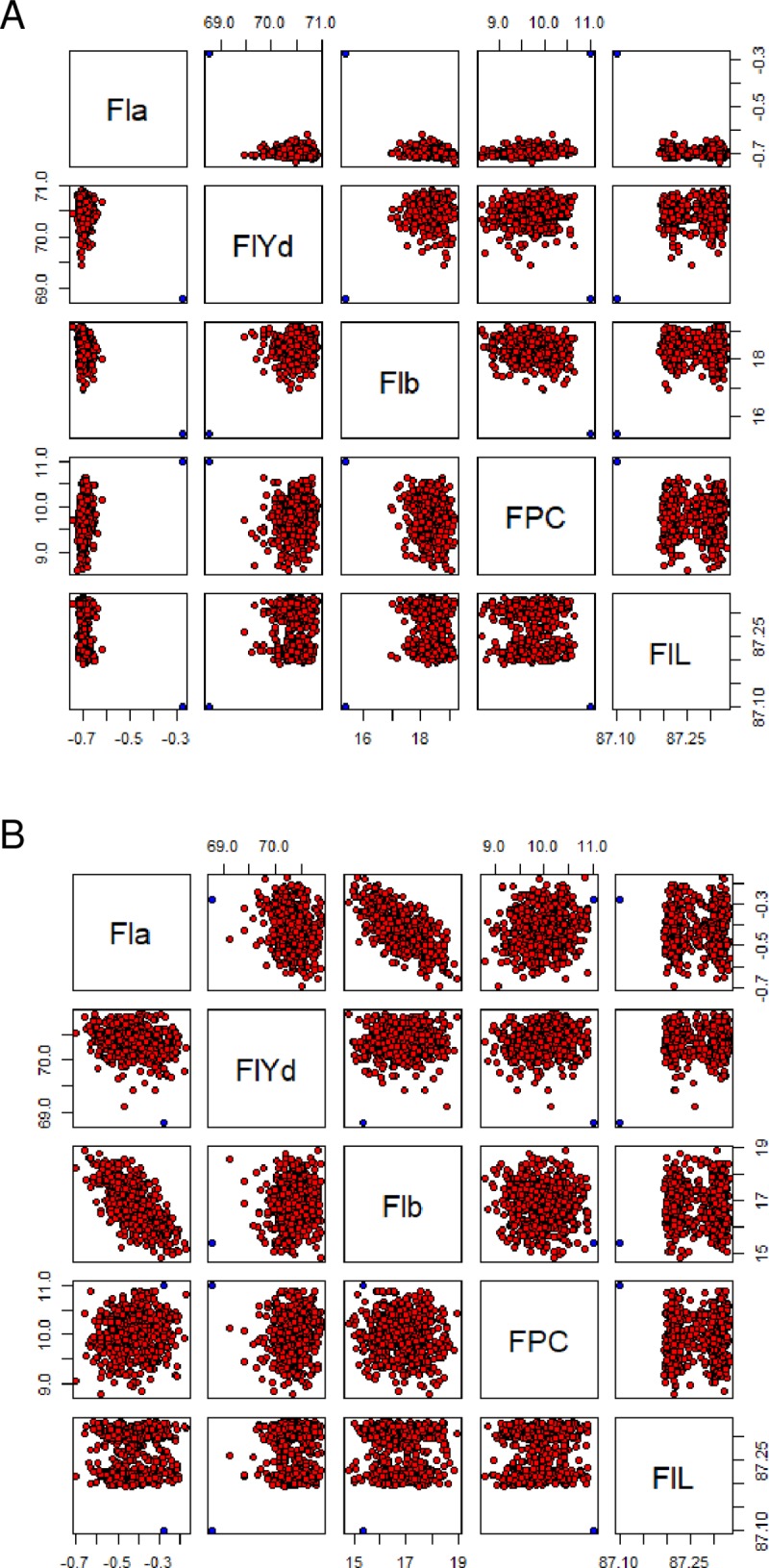
Scatter diagrams of predicted values for the five traits. Predicted values of derivatives from Ideotype 1 (A) and Ideotype 8 (B). Points in blue indicate Kitahonami’s values.

Based on the results of multifamily analysis, we selected an ideal genotype (Ideotype 1) that pyramided favorable alleles in terms of 21 regions containing FlYd and Fla QTLs (Table 6). Among possible derivatives of the ideotype, individuals with higher FlYd and lower Fla values than those measured for Kitahonami were observed, which indicated the possibility of breeding varieties with higher quality than Kitahonami (Fig 4A). However, the derivatives tended to show high Flb and low FPC values due to strong negative and moderate positive correlations between Fla and Flb and between Fla and FPC, respectively (Fig 2). Since Flb and FPC largely affect the end-use quality of flour, nine other ideotypes (Ideotypes 2–10) that differed in the number of fixed Fla QTL alleles were generated (Table 6). Scatter diagrams of the five traits indicated that variation in Flb and FPC increased as the number of fixed Fla QTLs decreased (Fig 4B, S2 Fig). The derivatives from Ideotypes 8–10 included individuals with higher FlYd and lower Fla values than those observed in Kitahonami and similar Flb and FPC values compared to those observed in this variety. Genotypes of derivatives from the ten ideotypes and their predicted phenotypes are given in S2 and S3 File, respectively.

**Table 6:** Genotypes of the ten ideotypes used for simulation.

		Position [cM]		Position [Mb]^1^			Ideotype^2^									
Region	Chr	Start	End	Start	End	QTL	1	2	3	4	5	6	7	8	9	10
1	1A	116.5	139.1	511.5	528.7	*QFla*.*m*.*tarc-1A*	A	A	A	A	A	A	A	A	A	-
2	2B	56.9	69.3	417.6	652.4	*QFla*.*m*.*tarc-2B*	B	B	-	-	-	-	-	-	-	-
3	2B	114.1	156.0	759.3	776.8	*QFlyd*.*m*.*tarc-2B*	B	B	B	B	B	B	B	B	B	B
4	2D	123.2	152.3	67.2	320.5	*QFlyd*.*m*.*tarc-2D*	B	B	B	B	B	B	B	B	B	B
5	3A	124.3	142.6	595.9	659.6	*QFlyd*.*m*.*tarc-3A*	A	A	A	A	A	A	A	A	A	A
6	3B	90.2	107.4	52.7	431.6	*QFlyd*.*m*.*tarc-3B*	B	B	B	B	B	B	B	B	B	B
7	4A	163.6	230.2	694.9	737.4	*QFla*.*m*.*tarc-4A*	B	B	B	B	B	B	-	-	-	-
8	4D	59.8	74.2	27.2	313.6	*QFlyd*.*m*.*tarc-4D*	B	B	B	B	B	B	B	B	B	B
9	5A	210.2	321.6	584.4	689.9	*QFlyd*.*m*.*tarc-5A*	A	A	A	A	A	A	A	A	A	A
10	5D	68.7	88.2	66.8	365.3	*QFla*.*m*.*tarc-5D*	B	B	B	B	B	B	B	-	-	-
11	5D	313.9	348.7	542.7	558.3	*QFlyd*.*m*.*tarc-5D*	B	B	B	B	B	B	B	B	B	B
12	6A	0.0	65.1	0.6	31.4	*QFla*.*m*.*tarc-6A*	B	B	B	B	-	-	-	-	-	-
13	6B	120.3	133.3	352.1	633.5	*QFlyd*.*m*.*tarc-6B*	B	B	B	B	B	B	B	B	B	B
14	6B	140.9	188.3	645.5	694.1	*QFla*.*m*.*tarc-6B*	A	A	A	-	-	-	-	-	-	-
15	6D	68.4	179.9	23.4	436.9	*QFlyd*.*m*.*tarc-6D; QFla*.*m*.*tarc-6D*	B	B	B	B	B	B	B	B	B	B
16	7A	141.1	154.2	127.8	488.7	*QFlyd*.*m*.*tarc-7A; QFla*.*m*.*tarc-7A*.*1*	B	B	B	B	B	B	B	B	B	B
17	7A	298.6	314.7	724.1	733.4	*QFla*.*m*.*tarc-7A*.*2*	A	A	A	A	A	-	-	-	-	-
18	7B	8.1	64.7	5.9	64.7	*QFlyd*.*m*.*tarc-7B; QFla*.*m*.*tarc-7B*.*1*	B	B	B	B	B	B	B	B	B	B
19	7B	215.3	238.9	706.8	739.4	*QFla*.*m*.*tarc-7B*.*2*	A	A	A	A	A	A	A	A	-	-
20	7D	9.1	52.8	13.3	32.9	*QFla*.*m*.*tarc-7D*	B	-	-	-	-	-	-	-	-	-
21	7D	129.5	208.0	71.6	530.0	*QFlyd*.*m*.*tarc-7D*	A	A	A	A	A	A	A	A	A	A

^1^ Positions in the IWGSC Chinese Spring RefSeq v1 sequence [26].

^2^ Genotype 'A' and 'B' indicate fixed region for non-Kitahonami’s and Kitahinami’s allele, respectively, while '-' indicates unconstrained region.

## Discussion

Recently, high-density SNP arrays and genotyping-by-sequencing (GBS) have been widely used for genetic analyses [34–38]. However, when using a specific crossed population, a SNP array is not an efficient way to develop genetic maps because a large number of probes do not show any polymorphism between parents. Since GBS relies on randomly distributed polymorphic sites flanking restriction enzyme recognition sites, the tags of GBS tend to show distribution bias across samples and the genome. Therefore, GBS is not suitable for genome-wide surveys using biparental populations. In this study, we used a target amplicon sequencing approach to construct genetic maps of three DHL populations. Because the DHL populations shared Kitahonami as the paternal parent, the use of common polymorphic sites as markers to compare maps among populations was quite beneficial. Taking advantage of the amplicon sequencing approach, well-saturated genetic maps were effectively constructed by selecting suitable marker sets for the target population.

Performing quality evaluations at a late stage often results in ostensibly promising wheat lines with high yield and resistance to diseases that cannot be released due to poor end-use quality traits, such as weak performance in milling and processing properties. Because the evaluation of quality traits is a labor-intensive and time-consuming step, it is difficult to perform genetic analysis of these traits using a large segregating population. In addition, due to unexpected environmental conditions, missing data caused by insufficient amounts of harvested seeds for milling often occur. In this study, we did not evaluate a complete set of DHLs during the four-year trial. However, with data for commonly used varieties and lines, we calculated least square means across environments, which could then be used as genetic estimates for each line. These values showed reasonable distribution patterns that were predicted by the parental values (Fig 1). Furthermore, heritability estimates of the target traits, FlYd and Fla, were relatively high, indicating that these traits were mainly governed by genetic factors (Table 2). These results indicate that selection based on the detected QTLs at an early stage of breeding has a great impact on improving these two traits. Since least square means across environments enabled us to detect reliable QTLs, historical data collected in breeding programs, which often include missing values, could be used for genetic analysis with the same statistical procedure.

QTLs detected by multifamily analysis tended to show higher QTL intensities than those detected by single-population analysis (Table 3, Table 4). For FlYd, single-population analysis revealed three, six and six QTLs in KK, SK and TK, respectively, while 12 QTLs were detected by multifamily analysis. Based on the physical positions of flanking markers, QTLs detected by single-population analysis were included in those detected by multifamily analysis, except for *QFlyd*.*tk*.*tarc-2B*. Because the intensity of this QTL (0.382) barely exceeded the 5% threshold value (0.372), further research is necessary to confirm its existence. Three QTLs were detected only by multifamily analysis. Among them, *QFlyd*.*m*.*tarc-3A* and *QFlyd*.*m*.*tarc-6D* showed relatively low posterior probabilities compared to those of other QTLs, while *QFlyd*.*m*.*tarc-5D* showed a high posterior probability, indicating that the QTL was reliable. For Fla, QTLs detected by single-population analysis were also found by multifamily analysis, except for *QFla*.*sk*.*tarc-1B*. Multifamily analysis revealed six QTLs that could not be found by single-population analysis. Among these QTLs, *QFla*.*m*.*tarc-6A*, *QFla*.*m*.*tarc-6B* and *QFla*.*m*.*tarc-7B* showed quite high posterior probabilities. In this study, for both FlYd and Fla, the average posterior probabilities from multifamily analysis were higher than those from single-population analysis, meaning that multifamily analysis improved the power to detect QTLs by increasing the size of the population. These results support the effectiveness of multifamily analyses in detecting actual QTLs described in Hayashi and Iwata [26].

Genome-wide QTL analyses of quality traits in soft wheat revealed flour yield and flour color QTLs on almost all chromosomes [5, 7, 23, 39–41]. By association mapping studies, many MTAs have been identified for wheat quality parameters. For example, 15, 28, 25 and 32 MTAs for flour color L*, a*, and b* and yellow pigment content, respectively, were detected [25]. Recently, using soft winter wheat from the eastern region of the United States, significant MTAs for flour yield were found on chromosomes 1B, 2A and 2B [42]. Our previous association mapping approach revealed approximately 20 QTLs related to flour yield that accumulated during the process of breeding [22]. Although these genetic factors are useful for the wheat research community, it is difficult to apply the information in actual breeding selection. Generally, breeding is a process of pyramiding favorable alleles from various materials into an elite genotype, such as a leading variety. Therefore, it is important to obtain allelism information for QTLs detected among breeding materials. A nested association mapping (NAM) population developed by crossing one elite variety with various germplasms can be used to dissect genetic factors for agronomically important traits [43–45]. A NAM population can also reveal the genetic architecture of genome-wide recombination rate variation, which would be useful information for improving the efficiency of gene pyramiding [46]. Therefore, the DHLs used in this study are suitable materials for genetic analysis as well as breeding materials for pyramiding favorable alleles of quality traits.

The predictabilities of five traits were evaluated by six-fold cross-validation (Table 5). The prediction accuracies of the two target traits, FlYd and Fla, were approximately 0.5, and the highest values exceeded 0.6, indicating that prediction of these traits from genotypes was possible. However, the prediction accuracies of these traits varied among cross-validation sets. To determine the appropriate selection criteria of a training set, further research is necessary to investigate the relationship between genotypes of the training set and selection candidates. Ranks based on prediction accuracies corresponded to those based on heritabilities (Table 2). Because heritability is defined as the portion of phenotypic variance that can be explained by genotype, it is reasonable for a trait with high heritability to show a high prediction accuracy based on genotype. As expected, the low-heritability trait, FlL, was not predictable (0.144). This result was consistent with the result of our QTL analysis, which revealed only one FlL QTL, with a contribution of 0.061.

In a breeding program, it is important to maintain a balance between antagonistic traits [47, 48]. Among the traits investigated in this study, the two target traits had clear selection criteria: higher flour yield was better, and lower flour a* was better. On the other hand, other traits such as DH, Flb and FPC had optimal values corresponding to those for target cultivation areas and end-use products. Multitrait QTL analysis reveals whether or not correlated traits are governed by the same genetic factors. In this study, we detected collocation of QTLs on group 7 chromosomes. According to the IWGSC functional annotations (https://wheat-urgi.versailles.inra.fr/Seq-Repository/Annotations) and expression profiles (http://www.wheat-expression.com/) [49, 50], annotated high-confidence genes around the QTL clusters were investigated (S4 File). Average transcripts per million (tpm) values from 166 studies were used to assess the expression levels of these genes in grains. Based on human-readable descriptions, phytoene synthase on chromosome 7B (TraesCS7B02G482000) is one of the causal genes for flour color parameters [15]. Among the genes highly expressed in grain tissues, genes related to carbohydrate metabolism, such as “glucose-1-phosphate adenylyltransferase” (TraesCS7A02G287400), “pyrophosphate—fructose 6-phosphate 1-phosphotransferase subunit beta” (TraesCS7A02G198200), “1,4-alpha-glucan branching enzyme” (TraesCS7A02G251400, TraesCS7B02G472400 and TraesCS7B02G472500), “starch synthase” (TraesCS7A02G189000 and TraesCS7D02G117800) and “debranching enzyme 1” (TraesCS7D02G133100) are candidate genes in the QTLs because carbohydrates are a dominant component of grains and are known to affect its processing quality. Transcription factors such as Zinc finger, MADS-box, Myb and NAC are also possible candidates because they are involved in various seed developmental processes. On chromosome 7A, a gene annotated as “Flowering locus T/ Terminal flower 1-like protein” (TraesCS7A02G229400) showed high expression levels in grains. Many studies have demonstrated that the proteins encoded by Flowering locus T-like (FT-like) genes act not only as major mobile flowering signals in flowering plants but also participate in the regulation of diverse developmental processes [51]. Therefore, pleiotropic effects of the 7A QTLs may be due to this multifunctionality of FT-like genes. Further studies delimiting QTL intervals are necessary to identify causal genes in the clusters. Although it is unclear whether or not the causal genes of these QTLs are the same, breeding selection of these QTL regions should be performed carefully while considering the balance of traits. A simulation study of the ideotypes showed the possibility of breeding lines with higher flour yield than Kitahonami. This possibility was realized by excluding *QFlyd*.*m*.*tarc-3A*, *Qflyd*.*m*.*tarc-5A* and *Qflyd*.*m*.*tarc-7D*, for which the Kitahonami alleles had negative effects. For flour color, the design of ideal genotypes was not simple because Fla was correlated with Flb and FPC. A lower Fla, which indicates high noodle-making quality, inevitably leads to a high Flb and low FPC. Since the Flb and FPC of flour greatly affect its end-use quality, it is important to optimize these values. Simulation revealed that fine-tuning of genotypes would be essential to obtain genotypes with ideal phenotypic values.

Bayesian QTL analyses of single populations and multiple families followed by simulations revealed the possibility of breeding varieties superior to Kitahoanmi. Trait predictability is an attractive technology with the potential to significantly improve breeding efficiency. Genomic predictions using a high-density genotyping platform were performed for quality traits of durum wheat [21, 52]. Recently, the genomic predictabilities of 35 key traits were reported, which demonstrated the potential of genomic selection for wheat end-use quality [53]. In this report, the prediction accuracy and standard deviation of flour yield across four seasons were 0.56 ± 0.04 and 0.45 ± 0.09 when cross-validation was performed within material categories (panels) and across panels, respectively. These values were comparable to our prediction accuracies, even though the number of markers used in this study was small. In regular breeding programs, due to high costs, it is still difficult to routinely obtain high-density genotypic data from a large number of selection candidates. The cost-effective amplicon sequencing method described herein would be a suitable platform for selecting several dozens of trait-associated chromosomal regions simultaneously. Furthermore, to increase the chance pyramiding QTLs by crossing, the growth acceleration method called 'speed breeding' [54] may be an effective tool for establishing the ideal genotypes presented here. Therefore, genome-wide target selection combined with speed breeding is a next-generation breeding strategy for high-quality wheat.

## Supporting information

S1 Fig Distributions of x50, Sm, FPC and Fash for the three populations. (TIF)

S2 Fig Scatter diagrams of predicted phenotypes for the ten ideotypes. (MP4)

S1 File Genotypes of DHLs and genetic and physical positions of markers. (XLSX)

S2 File Genotypes of derivatives of the ten ideotypes. (XLSX)

S3 File Phenotypes of derivatives of the ten ideotypes. (XLSX)

S4 File List of annotated genes around the QTL clusters on group 7 chromosomes. (XLSX)

S1 Table List of amplicon sequence markers used in this study. (XLSX)

S2 Table Number of observations for each trait, environment and population. (XLSX)

S3 Table Phenotypic data for the ten traits investigated. (XLSX)

S4 Table Summary of phenotypic data for the ten traits. (XLSX)

S5 Table Analysis of variance results for the ten traits. (XLSX)

S6 Table Least square mean values of the ten traits investigated. (XLSX)

S7 Table Threshold of QTL intensity at the 5% level. (XLSX)

S8 Table List of QTLs detected by each population analysis. (XLSX)

S9 Table Distributions of QTL intensity obtained by multifamily analysis. (XLSX)

S10 Table List of QTLs detected by multifamily analysis. (XLSX)
