# Supplementary figures and images for: Multifamily QTL analysis and comprehensive design of genotypes for high-quality soft wheat
Source: PLoS One. 2020 Mar 11;15(3):e0230326. doi: 10.1371/journal.pone.0230326 (PMC7065826; doi:10.1371/journal.pone.0230326)

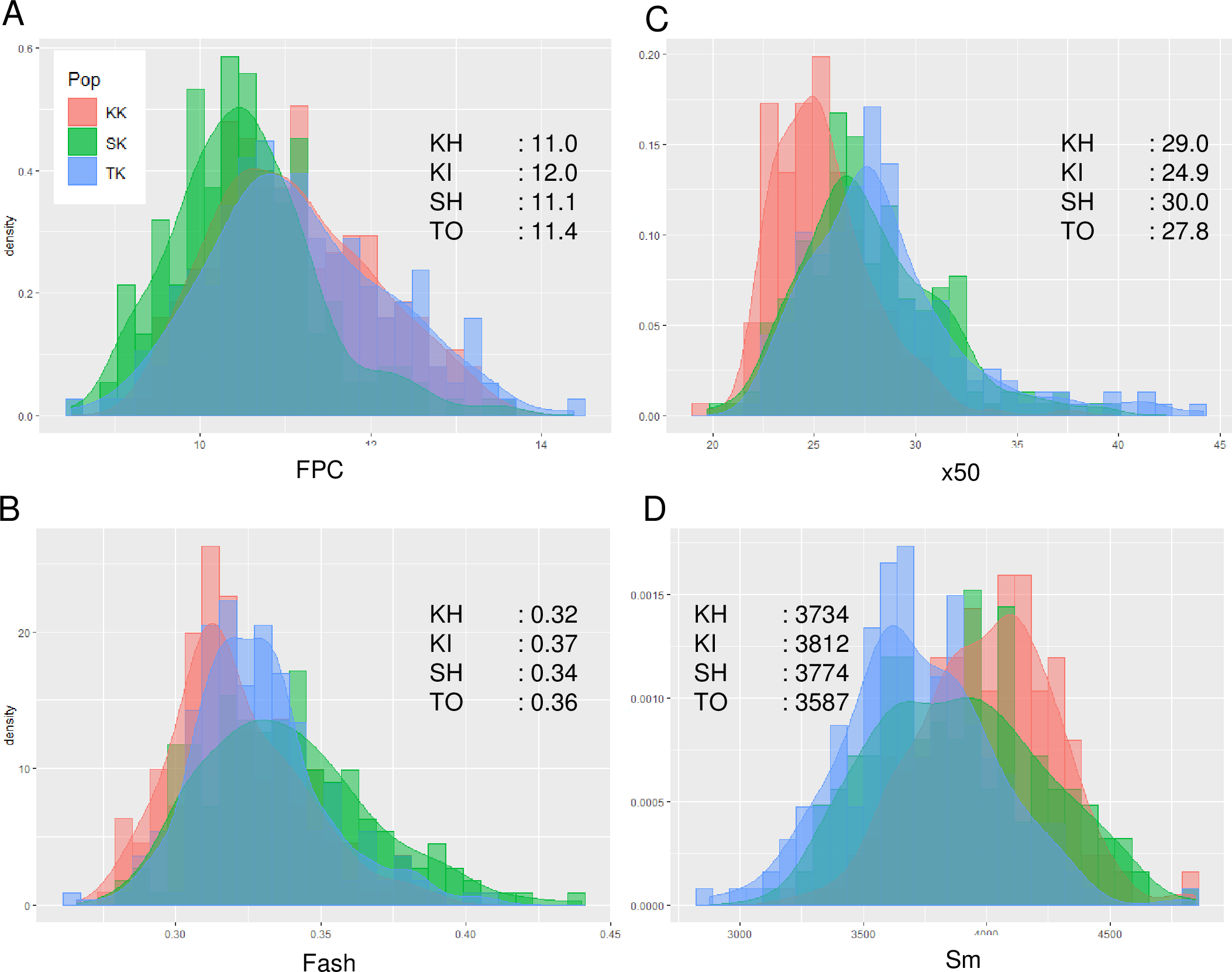

Supplement: S1 Fig — (TIF) [file pone.0230326.s001.tif]
